# Comorbidity and medication patterns in atrial fibrillation patients: association with adverse clinical outcomes

**DOI:** 10.1007/s11739-025-04047-6

**Published:** 2025-07-18

**Authors:** Dilek Celik, Cheima Amrouch, Søren Paaske Johnsen, Gregory Y. H. Lip, Davide Liborio Vetrano, Mirko Petrovic, Bruno Micael Zanforlini, Giuseppe Sergi, Nicola Ferri, Caterina Trevisan

**Affiliations:** 1 Department of Pharmaceutical and Pharmacological Sciences, University of Padua, Via Marzolo 5, 35131 Padua, Italy; 2 Department of Internal Medicine and Paediatrics, Ghent University, Ghent, Belgium; 3 Department of Public Health and Primary Care, Ghent University, Ghent, Belgium; 4 Danish Center for Health Services Research, Department of Clinical Medicine, Aalborg University, Aalborg, Denmark; 5 Liverpool Centre for Cardiovascular Science at University of Liverpool, Liverpool John Moores University and Liverpool Heart and Chest Hospital, Liverpool, UK; 6 Aging Research Center, Department of Neurobiology, Care Sciences and Society, Karolinska Institutet and Stockholm University, Stockholm, Sweden; 7 Stockholm Gerontology Research Center, Stockholm, Sweden; 8 Department of Medicine, University of Padua, Padua, Italy; 9 Department of Medical Sciences, University of Ferrara, Ferrara, Italy; 10 Veneto Institute of Molecular Medicine (VIMM), Padua, Italy

**Keywords:** Atrial fibrillation, Multimorbidity, Polypharmacy, Latent class analysis, Aging, Adverse events

## Abstract

Managing atrial fibrillation (AF) patients with multiple comorbidities and complex medications is challenging. This study aimed to identify different patient profiles of AF based on comorbidities and medicines combinations and to explore their associations with the risk of adverse outcomes. This cohort study included patients with AF from the anticoagulant outpatient clinic at a hospital in Italy, undergoing follow-ups every 6 months. Comorbidity and medication patterns were identified using latent class analysis. Cox regression was used to explore associations with thromboembolism, major bleeding, falls, and death—separately and composite. A total of 633 patients with AF (mean age 80.5 ± 6.9 years, 52.5% women) treated with direct oral anticoagulants were followed for a median of 24.2 (IQR 12.1–35.5) months. Four patterns were identified: unspecific pattern (39.0%), diabetes and liver pattern (14.8%), neurocognitive and psychiatric pattern (14.1%), and musculoskeletal, immunologic and dermatologic pattern (32.1%). After adjustments, the neurocognitive and psychiatric pattern was associated with a higher risk of the composite outcome (hazard ratio [HR] [95% CI]: 1.75 [1.56–3.82]), thromboembolism (HR: 3.04 [1.28–7.22]) and major bleeding (HR: 2.55 [1.05–6.22]) compared to the non-specific pattern. The musculoskeletal, immunologic, and dermatologic pattern was also associated with a higher bleeding risk (HR: 2.21 [1.05–4.65]). Stratified analyses showed that these links were stronger in patients without cancer, and there was significant interaction in bleeding risk based on cancer status (*p* = 0.014). Anticoagulated AF patients with the neurocognitive and psychiatric profile are at higher risk, emphasizing the need for holistic AF management.

**Supplementary Information:**

The online version contains supplementary material available at 10.1007/s11739-025-04047-6.

## Introduction

Atrial fibrillation (AF), which is the most common cardiac arrhythmia in older age, frequently occurs with multiple comorbidities, leading to a higher prevalence of concomitant drug use [1, 2]. Indeed, the prevalence of polypharmacy, defined as five or more concomitant drug use, in patients with AF ranges from 40 to 95%, depending on the setting and population considered [3–5].

Although implementation of direct anticoagulant (DOAC) treatment has improved outcomes in patients with AF, important risks remain to manage beyond anticoagulation, especially for patients with multiple long-term conditions [6]. The coexistence of multiple long-term conditions and the use of multiple medications in patients with AF have been associated with a higher risk of adverse outcomes in patients with AF [7], including major cardiovascular events, bleeding [8, 9], hospitalizations, falls, functional impairment [10], shorter survival, higher expenses for healthcare [11], and higher burden for patients and healthcare professionals [4]. Therefore, identifying patient characteristics based on both comorbidities and drugs that may impact clinical outcomes is critical.

By considering multiple diseases and drugs, advanced statistical techniques, such as latent class analysis (LCA), may improve risk stratification and provide a comprehensive view of how comorbidity and concurrent medications affect the risk of adverse outcomes, leading to more effective and safer AF management options. Indeed, counting the absolute number of diseases may not be sufficient to adequately assess the impact of multimorbidity. Psychosocial, biological, pharmacological, and additional health determinants may not only increase the propensity to develop individual comorbidities but also lead to their aggregation and potentially synergistic interaction [12]. In this context, clustering multimorbidity is helpful for identifying disease trends and combinations, as well as clinically complex phenotypes, which are linked with adverse health consequences [12, 13]. Moreover, by evaluating phenotypic patterns, healthcare providers can better tailor interventions and potentially reduce the negative consequences of complex disease combinations through better integrated care [14].

In the present study, we aimed to identify and characterise phenotypic patterns of older patients with AF based on their chronic comorbidities and medications and to assess the associations between these patterns and adverse clinical outcomes.

## Methods

The study was conducted according to the STrengthening the Reporting of OBservational studies in Epidemiology (STROBE) guidelines [15] (Supplementary Table 1).

### Study design and population

From the initial sample of 777 AF patients, we excluded 110 with missing values for the outcome events, 19 patients with no comorbidities, and 15 patients with no concomitant drug use, obtaining an analytical sample of 633 individuals. The final sample of this monocentric cohort study included 633 patients aged ≥ 65 years with AF who attended their first outpatient visit at the Geriatric Clinic at Padua University Hospital (Padua, Italy) from September 2013. All participants were receiving DOAC therapy based on current guidelines at baseline [16]. For this study, we considered only patients with AF who had at least one additional comorbidity and one additional medication apart from a DOAC.

The study complied with the ethical standards of the 1964 Declaration of Helsinki and its later amendments or comparable ethical standards and was approved by the local Ethical Committee (n. 5397/AO/22). All participants, or the next of kin for those with cognitive impairment, gave their written informed consent.

### Data collection

#### Baseline information

At their first outpatient visit, patients underwent a comprehensive geriatric assessment and a review of medical records, including recent blood examination, as part of the routine practice. For the present study, the following data were considered: sociodemographic and anthropometric information (age, sex, education; primary (5 years), middle school (8 years), high school (13 years), university or above (> 13 years), the presence of a caregiver, height, weight), alcohol consumption (yes/no), full blood count, serum creatinine, liver enzymes, and DOAC type. Mobility level was categorised as being independent, walking with aids or use of a wheelchair, and bedridden. From the above baseline information, thromboembolic (CHA_2_DS_2_-VASc score) [17] and bleeding risk (HAS-BLED score) [18] were calculated for each patient following the current recommendations.

The medical history of each patient was reviewed to assess the presence of comorbidities and concurrent medication use, from which a multidisciplinary team composed of a geriatrician, a pharmacologist, and a public health expert identified 17 disease and 18 drug groups (the complete list is reported in Supplementary Table 2).

#### Outcomes

Follow-up assessments were routinely conducted 1, 3, and 6 months after the first clinical visit and every 6 months thereafter. First, patients were contacted by phone by a clinical nurse, who conducted a structured interview with questions regarding any bleeding events, medication adherence, changes in the patient’s treatment regimen, emergency room visits or hospital admissions, and the occurrence of falls since the last contact. Second, patients were re-evaluated by a specialist physician, who checked the results of the phone interviews, new biochemical parameters assessments, and any clinical updates through medical and hospital records. For the present study, we considered data up to the 36-month follow-up on the outcomes reported below.

*Major Bleeding* was defined as a bleeding that needed hospital admission or invasive procedure or blood transfusion or causing a haemoglobin loss > 2 g/L or death.

*Thromboembolic events* included ischaemic stroke, transient ischemic attack (TIA), pulmonary embolism, deep vein thromboembolism, and other thromboembolisms (peripheral embolism, atrial thrombus, and left atrial appendage thrombus, etc.).

*All-cause death,* with data retrieved from medical and hospital records.

*Accidental falls* was defined according to the World Health Organization, as “an event which results in a person coming to rest inadvertently on the ground or floor or other lower level” [19].

The* composite outcome*, included incident thromboembolism, bleeding, and all-cause death, whichever occurred first.

### Statistical analysis

Patterns of chronic diseases and medications were identified through a latent class analysis using ‘*poLCA’* package in R, based on 18 drug and 17 disease groups described above. The ideal number of patterns was determined by evaluating the Bayesian Information Criterion (BIC) and the Akaike Information Criterion (AIC) (wherein lower values indicate a superior model fit [20]) as well as the distribution of latent classes in the study sample (Supplementary Table 3). The posterior probability of membership was calculated for all patients, and they were assigned to one of the patterns. Observed/Expected (O/E) ratios and exclusivity were calculated for each comorbidity and medication pattern to determine overrepresented disease and drug groups. O/E ratios were obtained by dividing disease and drug prevalence in the patterns by the disease and drug prevalence in the overall population. Exclusivity was defined as the proportion of patients with the disease included in the pattern over the total number of patients with the disease [21]. The comorbidity and medication patterns were defined as a combination of disease and drug use, with an O/E ratio of ≥ 2 and exclusivity of ≥ 25% (Supplementary 4).

Baseline characteristics of the participants were presented for the whole sample and by patterns. Binary and categorical variables were reported as frequencies (n) and percentages (%), and a chi-square test was used to compare frequencies. Continuous variables were reported as mean ± standard deviation (SD) or median and quartile range (IQR) if not near-normally distributed, and ANOVA or Kruskal–Wallis tests were used for the comparison.

For each pattern and outcome, we computed the incidence rate as the number of new events divided by the total person-time at risk, expressed as the number of events per 100 person-years. Unadjusted and adjusted Cox regression analysis was performed to test the risk of bleeding and thromboembolic events, falls, and the composite outcome as a function of the comorbidity and medication patterns. Multivariable adjusted Cox regression models were conducted to control for potential confounding factors. The covariates were identified based on the scientific rationale and current literature and included age, sex, education, and DOAC type for the composite outcome; education, DOAC type, and baseline CHA_2_DS_2_VASc score for the thromboembolic outcome; sex, education, DOAC type, and HAS-BLED score for the bleeding outcome; age, sex, education, DOAC type, stroke/TIA, and fall history for the fall outcome. Concerning the choice of confounders, age and sex were included since these sociodemographic factors have been associated with adverse outcomes in AF. Educational level may influence adherence to medication and self-management of chronic conditions. DOAC type was considered since anticoagulants may present differences in safety and efficacy characteristics. CHA₂DS₂-VASc and HAS-BLED scores are comprehensive tools for assessing, respectively, thromboembolic and bleeding risk. Finally, previous stroke/TIA and falls are strong predictors of future fall events. Collinearity between variables was evaluated while selecting the final set of confounders. Results were reported as hazard ratios (HR) with a 95% confidence interval (CI), and the overall level of statistical significance was designated at 5%.

In the regression analysis, missing values in categorical variables were managed by creating dummy variables, while individuals with missing data on continuous covariates were excluded from the final models.

The possible modifying effect of sex, age (considering the median values as a cut-off, i.e. 80 years), and mobility in the association between comorbidity and medication patterns and clinical outcomes was evaluated through the linear combinations of parameters (*lincom*) test. First, *lincom* test was performed to determine the interaction between potential modifying factors and comorbidity and medication patterns. Statistical significance was reported as 10% for the *lincom* test to identify interactions that may require further investigation. Second, analyses stratified by each potential effect modifier were performed. Moreover, given the potential effect of cancer on adverse clinical outcomes in AF patients [22], we performed a stratified analysis based on the presence of cancer status of patients to explore the impact of comorbidities and medication patterns in this subgroup.

The analyses were performed using R 4.3.3 (R Core Team 2020, Vienna, Austria), IBM SPSS Statistics (version 25), and Stata version SE 17 (StataCorp LLC, College Station, Texas, USA).

## Results

This monocentric cohort study included 633 patients with AF with a mean (SD) age of 80.5 (6.9) years (56.1% ≥ 80 years), and 333 (52.6%) were women (Table 1). Overall, 449 (70.9%) of patients had ≥ 3 diseases, and 519 (81.9%) were taking ≥ 3 drugs. Concerning mobility, 402 (63.5%) of patients were able to move independently, 176 (27.8%) required a walker or wheelchair, and 33 (5.2%) had more severe mobility impairments.

**Table 1:** Baseline characteristics of patients with AF based on the comorbidity and medication patterns

Variables	Total (n = 633)	Comorbidity and Medication Patterns			
		Unspecific pattern (n = 246)	Diabetes and Liver pattern (n = 94)	Neurocognitive and Psychiatric pattern (n = 90)	Musculoskeletal, Immunological and Dermatological pattern (n = 203)
*Female sex, n (%)*	333 (52.6)	121 (49.2)	49 (52.1)	49 (53.9)	114 (56.2)
*Age, mean (SD)*	81 (7)	80 (7)	79 (7)	84 (6)	81 (7)
*Age* ≥ *80, n (%)*	355 (56.0)	131 (53.3)	43 (45.7)	67 (74.2)	114 (56.2)
*Education, n (%)*					
Primary school	320 (50.1)	107 (46.5)	50 (56.8)	49 (63.6)	114 (60.3)
Middle school	97 (15.3)	45 (19.6)	17 (19.6)	11 (14.3)	24 (12.7)
High school	112 (17.7)	47 (20.4)	15 (17.1)	12 (15.6)	38 (20.1)
University or above	56 (8.7)	31 (13.5)	6 (6.8)	6 (6.5)	13 (6.9)
*Living arrangement, n (%)*					
Living alone	124 (20.0)	51 (21.5)	14 (15.1)	14 (15.1)	45 (22.5)
Living with family	460 (74.9)	176 (74.3)	72 (79.1)	65 (75.6)	147 (73.5)
Living in nursing home	24 (3.9)	7 (3)	3 (3.3)	8 (9.0)	6 (3.0)
Living in shared accommodations	7 (1.1)	3 (1.3)	2 (2.2)	0	2 (1.0)
*Mobility, n (%)*					
Independent	402 (63.5)	179 (72.8)	56 (59.6)	26 (29.2)	141 (69.5)
Moves with walking aids	136 (21.5)	47 (19.1)	22 (23.4)	22 (24.7)	45 (22.2)
Moves with wheelchair	40 (6.3)	7 (2.9)	8 (8.5)	18 (19.1)	7 (3.5)
Bedridden	33 (5.2)	4 (1.6)	3 (3.2)	19 (21.4)	7 (3.5)
*Presence of a caregiver, n (%)*	262 (41.2)	74 (30.1)	41 (43.6)	75 (83.2)	72 (35.5)
*Body Mass Index (kg/m*^*2*^*), n (%)*					
< 18.5	5 (0.8)	1 (0.4)	2 (2.1)	1 (1.1)	1 (0.5)
18.5–24.9	162 (25.6)	84 (34.2)	11 (11.7)	27 (30.3)	40 (19.7)
25–29.9	161 (25.3)	69 (28.1)	23 (24.5)	19 (20.2)	50 (24.6)
30–34.9	61 (9.7)	16 (6.5)	21 (22.3)	3 (3.4)	21 (10.3)
≥ 35	14 (2.2)	3 (1.2)	2 (2.1)	1 (1.1)	8 (3.9)
*History of fall, n (%)*	125 (19.6)	40 (16.3)	19 (20.2)	29 (31.5)	37 (18.2)
*Alcohol consumption, n (%)*	39 (6.2)	14 (5.7)	6 (6.4)	6 (6.7)	13 (6.4)
*History of stroke/TIA, n (%)*	160 (25.2)	60 (24.4)	18 (19.2)	39 (42.7)	43 (21.2)
*Total n. diseases, mean (SD)*	4.1 (1.5)	2.1 (1.0)	4.6 (1.7)	3.9 (1.6)	4.4 (1.5)
*Total n. drugs, mean (SD)*	4.1 (1.6)	2.9 (1.3)	4.5 (1.7)	4.9 (1.9)	3.9 (1.6)

N = 48 individuals had missing values on education, n = 18 in living arrangements, n = 22 in mobility, n = 19 in caregiver, n = 22 in mobility, n = 43 in fall history, n = 230 in Body Mass Index, and n = 4 in alcohol consumption. “Living with family” involves the number of patients living with relatives and formal care

**Fig. 1:**
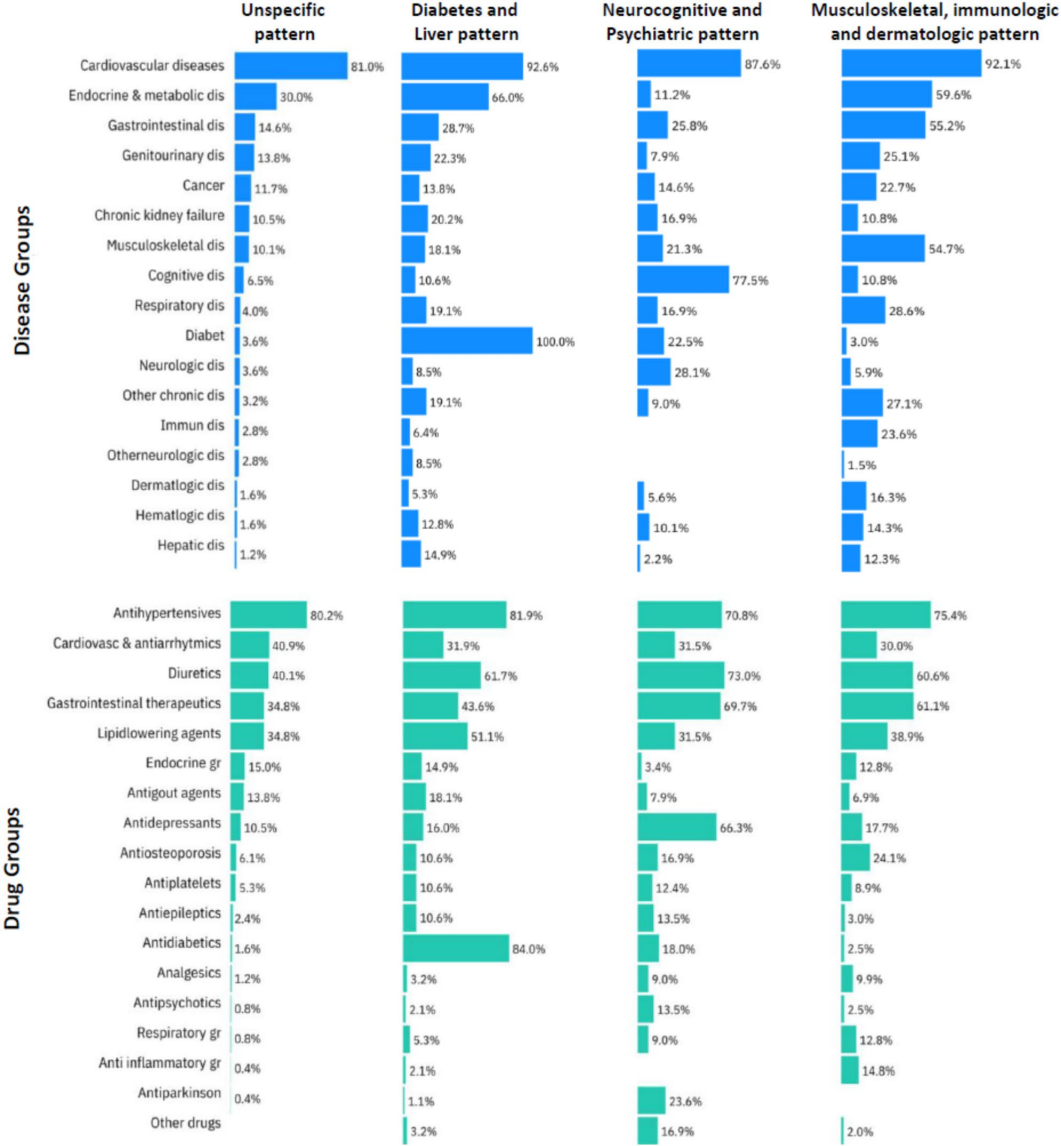
Proportions of disease and drug groups in the four comorbidity and medication patterns

Four comorbidity and medication patterns were identified (Fig. 1). No overrepresented disease or drug groups emerged in the first pattern, constituting an *unspecific pattern* and included 246 (39.0%) of patients. Three other patterns, based on the frequencies of comorbidities and medications, were identified: a *diabetes and liver pattern* (94 14.8%), a *neurocognitive and psychiatric pattern* (90, 14.1%), and a *musculoskeletal, immunological, and dermatological pattern* (203, 32.1%) (Supplementary Table 4).

Mobility and living arrangements varied among the four patterns, reflecting different levels of dependence (Table 1). Most patients in the unspecific pattern and musculoskeletal, immunological and dermatological pattern showed a high level of independent mobility (72.8% and 69.5%, respectively), and most were living with family (74.3% and 73.5%, respectively). The neurocognitive and psychiatric pattern exhibited the most compromised mobility, with the highest proportions of patients using walking aids (24.7%), wheelchairs (19.1%), or being bedridden (21.4%). The diabetes and liver pattern demonstrated moderate mobility limitations, with a mix of independently mobile patients (59.6%) and those requiring walking aids (23.4%) or wheelchairs (8.5%).

The most frequently prescribed DOAC in our sample was rivaroxaban (37.7%), followed by dabigatran (26.9%), apixaban (24.0%), and edoxaban (11.6%) (Table 2). The CHA_2_DS_2_VASc score suggested that all comorbidity and medication patterns were at high risk of stroke: 75.3% had a CHA_2_DS_2_VASc score ≥ 2. A high risk of bleeding (HAS-BLED score ≥ 3) was observed more frequently in the diabetes and liver pattern (41.5%), neurocognitive and psychiatric pattern (39.3%), and musculoskeletal, immunologic, and dermatologic pattern (40.9%). Previous falls were reported by 19.6% and one-quarter had a history of stroke or TIA (Table 1).

**Table 2:** Atrial fibrillation-related information and biochemical parameters of patients at baseline

	Total (n = 633)	Comorbidity and Medication Patterns			
		Unspecific pattern (n = 246)	Diabetes and Liver pattern (n = 94)	Neurocognitive and Psychiatric pattern (n = 90)	Musculoskeletal, Immunological and Dermatological pattern (n = 203)
*DOAC use, n (%)*					
Dabigatran	170 (26.9)	86 (35.0)	20 (21.3)	15 (16.9)	49 (24.1)
Rivaroxaban	238 (37.7)	83 (33.7)	34 (36.2)	45 (50.6)	76 (37.4)
Apixaban	152 (24.0)	48 (19.5)	31 (33.0)	24 (25.8)	49 (24.1)
Edoxaban	73 (11.6)	29 (11.8)	9 (9.6)	6 (6.7)	29 (14.3)
*DOAC dosage, n (%)*					
Low	416 (65.8)	163 (66.3)	50 (53.2)	69 (77.5)	134 (66.0)
High	217 (34.2)	83 (33.7)	44 (46.8)	21 (22.5)	69 (34.0)
*Serum creatinine (mg/dl), mean (SD)*	0.95 (0.29)	0.95 (0.28)	1.00 (0.27)	0.93 (0.27)	0.94 (0.32)
*Clearance creatinine (cc/min), mean (SD)*	62.3 (22.3)	61.95 (21.3)	65.69 (24.2)	56.46 (18.85)	63.77 (23.7)
*AST (U/l), mean (SD)*	24 (11)	25 (11)	21 (8)	22 (9)	24 (12)
*ALT (U/l), mean (SD)*	20 (12)	22 (14)	20 (10)	17 (9)	20 (11)
*Red blood count (*× *10*^*3*^*/µl), mean (SD)*	4495 (1982)	4552 (571)	4315 (670)	4155 (704)	4653 (3333)
*Haemoglobin (g/l), mean (SD)*	132.2 (16.9)	137.5 (15.7)	129.3 (16.5)	124.8 (17.1)	130.3 (16.5)
*CHA*_*2*_*DS*_*2*_*-VASc, mean (SD)*	2.52 (1.4)	2.14 (1.3)	3.40 (1.2)	2.97 (1.5)	2.37 (1.3)
*Risk category, n (%)*					
High (≥ 2), n (%)	477 (75.3)	161 (65.5)	92 (97.9)	74 (82.0)	150 (73.9)
Moderate (1), n (%)	130 (20.6)	67 (27.2)	2.(2.1)	13 (14.6)	48 (23.7)
Low (0), n (%)	26 (4.1)	18 (7.3)	0.00	3 (3.4)	5 (2.5)
*HAS-BLED Score, mean (SD)*	2.12 (1.1)	1.87 (1.1)	2.35 (1.0)	2.29 (1.1)	2.23 (1.0)
*Risk category, n (%)*					
Very high (> 5), n (%)	1 (0.2)	0	0	1 (1.1)	0
High (3–5), n (%)	220 (34.8)	63 (25.6)	39 (41.5)	35 (39.3)	84 (40.9)
Moderate (2), n (%)	238 (37.7)	94 (38.2)	37 (39.4)	33 (37.1)	74 (36.5)
Low (0–1), n(%)	173 (27.4)	89 (36.2)	18 (19.2)	20 (22.5)	46 (22.7)

*Abbreviations*. *DOAC* direct anticoagulant, *ALT* alanine aminotransferase, *AST* aspartate aminotransferase

### Incident rates of adverse events in the comorbidity and medication patterns

The total median follow-up was 24.2 (IQR 12.1–35.5) months. Table 3 shows the incidence rates of the outcomes per 100 person-years (p-y) for each comorbidity and medication pattern. During the follow-up period, the incidence rate of composite outcome was 1.0 per 100 p-y (95% CI, 0.9–1.2), while thromboembolic events occurred at 0.3 per 100 p-y (95% CI, 0.2–0.4). The incidence rate of bleeding events was 0.4 per 100 p-y (95% CI, 0.3–0.5) and falls was 1.6 per 100 p-y (95% CI, 1.4–1.8). The incidence rate of death outcome was 5.3 per 100 p-y (95% CI, 4.2–6.7). Notably, apart from death event, the neurocognitive and psychiatric pattern had the highest incidence rates (per 100 p-y) of composite, thromboembolic, bleeding, and fall outcomes compared to the other patterns.

**Table 3:** Overall number of adverse outcomes and incident rates in AF patients by comorbidity and medication pattern

Outcomes	Total events	Number of events in each pattern	Median FU (IQR)	Incidence rates	Incidence rates for each pattern per 100 p-y (CI)
Composite outcome	140	UN = 46 DL = 20 NCP = 33 MID = 41	22.6 (11.6–35.2)	1.0 (0.9–1.2)	UN = 0.8 (0.6–1.1) DL = 0.9 (0.6–1.5) NCP = 1.8 (1.3–2.6) MID = 1.0 (0.7–1.3)
Thromboembolic outcome	44	UN = 13 DL = 5 NCP = 10 MID = 16	23.7 (12.0–35.4)	0.3 (0.2–0.4)	UN = 0.2 (0.1–0.4) DL = 0.2 (0.1–0.5) NCP = 0.5 (0.3–1.0) MID = 0.4 (0.2–0.6)
Bleeding outcome	57	UN = 14 DL = 9 NCP = 12 MID = 22	24.0 (12.0–35.4)	0.4 (0.3–0.5)	UN = 0.2 (0.1–0.4) DL = 0.4 (0.2–0.8) NCP = 0.6 (0.3–1.1) MID = 0.5 (0.3–0.7)
Fall outcome	194	UN = 76 DL = 25 NCP = 28 MID = 65	17.7 (11.0–33.1)	1.6 (1.4–1.8)	UN = 1.5 (1.2–1.9) DL = 1.3 (0.9–1.9) NCP = 1.9 (1.3–2.8) MID = 1.7 (1.3–2.2)
Death outcome	72	UN = 28 DL = 11 NCP = 22 MID = 10	19.8 (11.2–26.3)	5.3 (4.2–6.7)	UN = 4.9 (3.4–7.0) DL = 5.4 (3.0–9.7) NCP = 5.4 (3.5–8.3) MID = 5.8 (3.1–10.8)

*Abbreviations*. *UN* unspecific pattern, *DL* diabetes and liver pattern, *NCP* neurocognitive and psychiatric disease pattern, *MID* musculoskeletal, immunologic and dermatologic pattern, *FU* follow-up *IQR* interquartile range, *p-y* person year, *CI* confidence interval

### Outcome occurrence across comorbidity and medication patterns

Unadjusted and adjusted Cox regression models for the adverse outcomes as a function of comorbidity and medication patterns are reported in Table 4. The neurocognitive and psychiatric pattern was associated with a higher hazard of the composite outcome (adjusted HR [95% CI]: 1.75 [1.06–2.90]) and thromboembolic events (aHR [95% CI]: 3.04 [1.28–7.22]) compared with the unspecific pattern. Moreover, the risk of bleeding was significantly increased in the neurocognitive and psychiatric, and the musculoskeletal, immunologic, and dermatological patterns (aHR [95% CI]: 2.55 [1.05–6.22]; aHR [95% CI]: 2.21 [1.05–4.65], respectively) compared to the unspecific pattern.

**Table 4:** Unadjusted and adjusted hazard ratios of adverse clinical outcomes in AF patients by comorbidity and medication pattern

Outcomes	Hazard ratios (95%CI)	Comorbidity and medication patterns						
		Unspecific pattern	Diabetes and liver pattern	p-value	Neurocognitive and psychiatric pattern	p-value	Musculoskeletal, immunological, and dermatological pattern	p-value
Composite outcome	Unadjusted HR (95% CI)	Ref	1.20 (0.71–2.03)	0.497	**2.44 (1.56–3.82)**	** < 0.001**	1.23 (0.80–1.87)	0.344
	Adjusted HR (95% CI)	Ref	1.57 (0.90–2.75)	0.111	**1.75 (1.06–2.90)**	**0.029**	1.30 (0.83–2.05)	0.250
Thromboembolic outcome	Unadjusted HR (95% CI)	Ref	1.04 (0.37–2.93)	0.934	**2.51 (1.10–5.73)**	**0.029**	1.62 (0.78–3.38)	0.194
	Adjusted HR (95% CI)	Ref	1.28 (0.44–3.78)	0.650	**3.04 (1.28–7.22)**	**0.012**	1.73 (0.70–3.80)	0.173
Bleeding outcome	Unadjusted HR (95% CI)	Ref	1.76 (0.76–4.07)	0.185	**2.84 (1.31–6.14)**	**0.008**	**2.09 (1.07–4.09)**	**0.031**
	Adjusted HR (95% CI)	Ref	2.04 (0.83–5.03)	0.121	**2.55 (1.05–6.22)**	**0.039**	**2.21 (1.05–4.65)**	**0.037**
Fall outcome	Unadjusted HR (95% CI)	Ref	0.83 (0.53–1.31)	0.432	1.27 (0.83–1.97)	0.274	1.16 (0.83–1.61)	0.389
	Adjusted HR (95% CI)	Ref	1.12 (0.70–1.80)	0.632	0.97 (0.60–1.55)	0.887	1.25 (0.88–1.78)	0.216

Bold values depicts statistically significant results at p < 0.05 level

Adjusted models for each outcome: *composite outcome*: age, sex, education, direct anticoagulant type; *thromboembolic outcome*: education, direct anticoagulant type, CHA_2_DS_2_VASc; *bleeding outcome*: sex, education, direct anticoagulant type, HASBLED Score; *fall outcome*: age, sex, education, direct anticoagulant type, stroke/TIA, fall history

*CI* confidence Intervals, *Ref* reference, *HR* hazard ratio

#### Subgroup analyses

When testing the possible modifying effect by age, sex, mobility level, and cancer status (Supplementary Table 5), no significant interactions were evident for the first two factors. However, the association between the neurocognitive and psychiatric pattern and composite, and thromboembolic outcomes were statistically significant in women and in those aged ≥ 80 years. Considering mobility, dependent patients within the neurocognitive and psychiatric pattern had a higher risk of the composite outcome (aHR [95% CI]: 2.59, [1.27–5.30]), bleeding (aHR [95% CI]: 10.99 [1.34–90.19]), and thromboembolic events (aHR [95% CI]: 5.19 [1.08–24.90]). Patients who were independently mobile and belonged to the neurocognitive and psychiatric pattern had an elevated risk of falls (aHR [95% CI]: 1.97 [1.02–3.79]). For the cancer status, we observed a significant interaction between patients with cancer and those without cancer in the musculoskeletal, immunologic, and dermatologic pattern concerning the risk of bleeding events (interaction p-value = 0.014). Additionally, we performed Cox models stratified by cancer status (Supplementary Table 6) to evaluate if the cancer status may alter the association between comorbidity and medication patterns and adverse outcomes. The findings indicated that the neurocognitive and psychiatric pattern and the musculoskeletal, immunologic, and dermatologic pattern were associated with increased risk of thromboembolic events in patients free from cancer (aHR [95% CI]: 3.50 [1.27–9.62], 3.59 [1.47–8.74], respectively). Moreover, these patterns and the Diabetes and liver pattern were associated with a higher risk of bleeding (aHR [95% CI]: 4.21 [1.54–11.47], 3.62 [1.48–8.84], 3.05[1.09–8.55], respectively).

## Discussion

In this study, we examined the associations between comorbidity, concurrent medications, and adverse outcomes in a cohort of AF outpatients treated with DOACs. We identified four comorbidity and medication patterns, among which the neurocognitive and psychiatric pattern was associated with a higher risk of thromboembolic events. Moreover, an increased risk of bleeding was observed in both the neurocognitive and psychiatric and the musculoskeletal, immunologic, and dermatologic patterns.

In previous studies in a variety of populations and clinical settings, comorbidity patterns showed a differential association with several health outcomes, including major adverse cardiovascular events, thromboembolism, major bleeding, quality of life, and healthcare utilization [8, 23–25]. The four comorbidity and medication patterns detected in our cohort of older outpatients share some features with those identified in other populations. Prior studies also found a clinically complex pattern of patients where neuropsychiatric conditions tended to cluster together [12, 26–29].

Our findings indicate that patients with neurocognitive and psychiatric comorbidities carry the highest burden of adverse outcomes, aligning with the existing literature on AF patients [30]. Indeed, recent data suggest that cognitive dysfunction is a significant risk factor for stroke, complicating the treatment of anticoagulant therapy in AF patients [31]. In particular, patients with AF (72.5% taking DOAC therapy) with severe cognitive impairment showed worse clinical outcomes and a higher risk of all-cause death, stroke/systemic embolism, bleeding, and heart failure [32]. Moreover, mental health conditions such as depression, anxiety, and psychotic disorders have been linked to lower DOAC adherence, increasing stroke and bleeding risk in AF patients [33]. Also, concurrent use of antiepileptics and DOAC drugs may increase the risk of thromboembolic events [34]. These findings, overall, are in line with our results and suggest that AF patients treated with anticoagulants and presenting neurocognitive and psychiatric comorbidities may be more vulnerable to both thromboembolic and bleeding events. Additionally, the higher risk of bleeding observed in the musculoskeletal, immunologic, and dermatologic pattern may be attributed to the higher prevalence of gastrointestinal diseases and the use of anti-inflammatory drugs within this group, both known to increase bleeding risk in AF patients on anticoagulant therapy [35–37]. Of note, any bleeding is associated with a higher risk of adverse clinical outcomes and a greater risk of oral anticoagulation discontinuation [38].

In addition to the detrimental impact of neurocognitive and psychiatric disorders on AF-related adverse outcomes, this pattern potentially represents vulnerable as well as the most clinically complex patients, characterized by a high medication burden compared to their comorbidities. Patients in the neurocognitive and psychiatric patterns had the highest number of drugs per comorbidity, implying that this group may have more severe chronic conditions and higher drug burden than the other patterns. Moreover, these patients were more likely to be functionally impaired, supporting the evidence that frailty, disability, and neurocognitive disorders often overlap and influence each other [39]. Although DOAC therapy may benefit several health-related outcomes [40], the current literature suggests that their efficacy and safety may vary with frailty levels, requiring patient-centred treatment decisions [41, 42]. Frailty is more prevalent in patients with multiple comorbidities and has been associated with a higher risk of bleeding [43]. In keeping with these findings, we also observed a higher risk of both bleeding and thromboembolic events in the subgroup of AF patients with neurocognitive and psychiatric disorders and the most severe mobility impairments. This confirms the challenges of managing AF due to coexistent conditions such as cognitive impairment, frailty, and polypharmacy [44]. Detecting patients with a higher bleeding risk is crucial since the management of these individuals can be optimized by closely monitoring their medical conditions and involving them in preventive interventions to avoid events that may predispose bleeding events (e.g., improving patient education, self-management, and medication adherence, correcting risk factors of falls).

Considering the combinatorial risks of AF, comorbidities, and medications vs falls [44], we evaluated whether the identified patterns were associated with such an outcome. Interestingly, none of the comorbidity and medication patterns was independently associated with increased fall risk. This finding contrasts with previous studies suggesting that AF patients with neurocognitive and psychiatric comorbidities, particularly those using antipsychotics, antidepressants, and benzodiazepines, and those with cognitive impairment, are at higher risk of injurious falls [45–48]. In addition, anti-Parkinson medication use may also increase the risk of falls due to orthostatic hypotension [49, 50]. However, when interpreting and comparing our results with those of previous studies, one should consider that we did not account for the dosage and specific medications used. Indeed, while psychotropic agents are generally associated with higher fall risk, differences in dosage, duration, or even medication type (e.g., newer antipsychotics or antidepressants) could have influenced this risk in our cohort. Finally, in our study, the detrimental effect of some hypotensive medications could have been mitigated because patients were under medical monitoring due to AF, with possible adjustment of chronic treatments or risk behaviours.

Of note, the subgroup analysis showed an increased fall risk only among AF patients in the neurocognitive and psychiatric pattern who were independently mobile. This result suggests that the impact of these disorders and medications on the risk of falls is strongly influenced by mobility status, affecting those who move more and, probably, in a less cautious way. Indeed, cognitive and psychiatric disorders may influence the individuals’ capacity to detect potentially harmful situations and can make fear of falling a maladaptive response that does not prevent but rather increases the probability of falling [51]. Identifying older AF patients at higher risk of falls based on their comorbidity and medication patterns is critical to planning appropriate preventive interventions (e.g., correction of environmental risk factors, regular medication review, education, and balance/resistance training). However, it is now largely recognized that this aspect should not contraindicate to anticoagulation since DOAC use has been demonstrated to be safe also in older adults with a history of falls, with a relatively low bleeding risk outweighed by the benefit in terms of stroke risk reduction [52]. Our findings highlight the importance of moving beyond single disease-drug associations towards understanding complex, real-world multimorbidity profiles. Informatics-based decision-support tools could help clinicians identify high-risk patients using routinely collected data, ultimately improving risk stratification, follow-up planning, and preventive care in older adults with AF. Given our findings, identifying a patient as belonging to of musculoskeletal or neurocognitive pattern may warrant closer follow-up or geriatric screening.

### Strengths and limitations

This study has several strengths. Using real-world data reflects clinical practice and patient outcomes with less bias than from selected cohorts or restricted registry data. The availability of detailed functional status data, which is usually not captured in national registries, allows a broader evaluation of patient health and the impact of functional impairments on outcomes. In addition, the relatively large sample size of older adults strengthens the validity of the conclusions, especially for this high-risk population. Finally, considering accidental falls, including those that did not result in hospitalisation, allows for a more comprehensive evaluation of fall risk, which is critical for understanding the whole range of fall-related morbidity in older people.

While our research yields valuable insights, several limitations must be addressed. First, our study is monocentric and possible selection bias could have occurred since patients accessing the outpatient clinic are more likely to be the healthiest ones. These limitations, overall, may have limited the sample representativeness of the general AF older population. Second, the recording of fall events relies on self-reported data; therefore, it can be affected by recall bias, especially given the advanced age of the population. Third, we did not account for comorbidities or concurrent drug use that emerged during the follow-up, restricting our ability to examine the longitudinal impact of these factors adequately. Fourth, more extended follow-ups are required to explore the outcomes of AF patients in the longer term, considering the varying comorbidities and medication patterns over time. Finally, some analyses had limited statistical power due to the small number of patients within certain comorbidity and medication patterns who experienced the outcomes of interest; therefore, these results should be interpreted with caution. Given the small number of patients with cancer, we were unable to evaluate the impact of comorbidity and medication patterns on adverse outcomes within this specific subgroup. Moreover, although our analyses were adjusted for several covariates, potential confounding could still exist.

## Conclusion

Anticoagulated patients with AF and associated neurocognitive and psychiatric comorbidities are at higher risk of adverse clinical events, including thromboembolism and bleeding. These findings suggest the need for a comprehensive evaluation of AF patients with specific comorbidities and medication patterns and a more holistic or integrated care approach to their management. Implementation of findings in clinical practice would involve the evaluation of comorbidities and drug patterns through informatic tools, which may help detect patients with a higher risk of adverse clinical outcomes.

## Supplementary Information

Below is the link to the electronic supplementary material.

Supplementary file1 (DOCX 61 KB)

## Data Availability

Data will be available upon reasonable request to Dr. Bruno Micael Zanforlini via email.
