# Supplementary material for: Comorbidity and medication patterns in atrial fibrillation patients: association with adverse clinical outcomes
Source: Intern Emerg Med. 2025 Jul 18;20(7):2029–40. doi: 10.1007/s11739-025-04047-6 (PMC12534355; doi:10.1007/s11739-025-04047-6)
Supplement: Supplementary file 1 — Supplementary file1 (DOCX 61 KB) [file 11739_2025_4047_MOESM1_ESM.docx]

**SUPPLEMENTARY MATERIAL**

## **Supplementary Table 1.** Compliance to the STROBE (Strengthening the Reporting of Observational Studies in Epidemiology) reporting guideline

|  | **Item No.** | **Recommendation** | **Page No.** |
| --- | --- | --- | --- |
| **Title and abstract** | 1 | (*a*) Indicate the study’s design with a commonly used term in the title or the abstract | 1 |
|  |  | (*b*) Provide in the abstract an informative and balanced summary of what was done and what was found | 2 |
| **Introduction** | | |  |
| Background/rationale | 2 | Explain the scientific background and rationale for the investigation being reported | 3 |
| Objectives | 3 | State specific objectives, including any prespecified hypotheses | 3 |
| **Methods** | | |  |
| Study design | 4 | Present key elements of study design early in the paper | 4 |
| Setting | 5 | Describe the setting, locations, and relevant dates, including periods of recruitment, exposure, follow-up, and data collection | 4,5 |
| Participants | 6 | (*a*) Give the eligibility criteria, and the sources and methods of selection of participants. Describe methods of follow-up | 4,5 |
|  |  | (*b*) For matched studies, give matching criteria and number of exposed and unexposed | / |
| Variables | 7 | Clearly define all outcomes, exposures, predictors, potential confounders, and effect modifiers. Give diagnostic criteria, if applicable | 4,5 |
| Data sources/ measurement | 8* | For each variable of interest, give sources of data and details of methods of assessment (measurement). Describe comparability of assessment methods if there is more than one group | 4-6 |
| Bias | 9 | Describe any efforts to address potential sources of bias | 13,14 |
| Study size | 10 | Explain how the study size was arrived at | 4 |
| Quantitative variables | 11 | Explain how quantitative variables were handled in the analyses. If applicable, describe which groupings were chosen and why | 4-6 |
| Statistical methods | 12 | (*a*) Describe all statistical methods, including those used to control for confounding | 6 |
|  |  | (*b*) Describe any methods used to examine subgroups and interactions | 7 |
|  |  | (*c*) Explain how missing data were addressed | 7 |
|  |  | (*d*) If applicable, explain how loss to follow-up was addressed | / |
|  |  | (*e*) Describe any sensitivity analyses | / |
| **Results** | | |  |
| Participants | 13* | (a) Report numbers of individuals at each stage of study—eg numbers potentially eligible, examined for eligibility, confirmed eligible, included in the study, completing follow-up, and analysed | 8 |
|  |  | (b) Give reasons for non-participation at each stage | / |
|  |  | (c) Consider use of a flow diagram | / |
| Descriptive data | 14* | (a) Give characteristics of study participants (eg demographic, clinical, social) and information on exposures and potential confounders | 8,Table 1 & 2 |
|  |  | (b) Indicate number of participants with missing data for each variable of interest | Table 1 & 2 |
|  |  | (c) Summarise follow-up time (eg, average and total amount) | 9,Table 3 |
| Outcome data | 15* | Report numbers of outcome events or summary measures over time | 9, Table 3 |
| Main results | 16 | (*a*) Give unadjusted estimates and, if applicable, confounder-adjusted estimates and their precision (eg, 95% confidence interval). Make clear which confounders were adjusted for and why they were included | 9,10, Table 4 |
|  |  | (*b*) Report category boundaries when continuous variables were categorized | 8,9, Figure 1, Supplementary Table 4 |
|  |  | (*c*) If relevant, consider translating estimates of relative risk into absolute risk for a meaningful time period | / |
| Other analyses | 17 | Report other analyses done—eg analyses of subgroups and interactions, and sensitivity analyses | 9,10, Supplementary Table 5 & 6 |
| **Discussion** | | |  |
| Key results | 18 | Summarise key results with reference to study objectives | 10-14 |
| Limitations | 19 | Discuss limitations of the study, taking into account sources of potential bias or imprecision. Discuss both direction and magnitude of any potential bias | 13,14 |
| Interpretation | 20 | Give a cautious overall interpretation of results considering objectives, limitations, multiplicity of analyses, results from similar studies, and other relevant evidence | 10-14 |
| Generalisability | 21 | Discuss the generalisability (external validity) of the study results | 13,14 |
| **Other information** | | |  |
| Funding | 22 | Give the source of funding and the role of the funders for the present study and, if applicable, for the original study on which the present article is based | 15 |

*Give information separately for exposed and unexposed groups.

**Supplementary Table 2.** List of groups of chronic diseases and medications recorded at baseline

| **Drug groups** | **Disease groups** |
| --- | --- |
| Antihypertensives | Respiratory diseases |
| Lipid lowering agents | Cardiovascular diseases |
| Cardiovascular/antiarrhythmics* | Endocrine & metabolic diseases |
| Diuretics | Neurologic diseases |
| Gastrointestinal therapeutics | Cognitive diseases |
| Respiratory drugs | Gastrointestinal diseases |
| Anti-Parkinson drugs | Hepatic diseases |
| Antiplatelets | Genitourinary diseases |
| Antidiabetics | Musculoskeletal diseases |
| Analgesics | Diabetes |
| Antigout agents | Haematologic diseases |
| Endocrine drugs | Immune disease |
| Antiepileptics | Dermatologic diseases |
| Antipsychotics | Cancer |
| Anti-inflammatory drugs | Chronic kidney failure |
| Antidepressants | Other chronic diseases (including sensory impairments) |
| Antiosteoporosis | Other neurological diseases |
| Other drugs |  |

* Cardiovascular/antiarrhythmic drug group included the use of digoxin, nitrates, amiodarone, diltiazem, verapamil, and other antiarrhythmics.

**Supplementary Table 3.** Bayesian and Akaike Information Criteria for the latent class models evaluated

|  | **Bayesian Information Criterion** | **Akaike Information Criterion** |
| --- | --- | --- |
| 2-class model | 17669.24 | 17353.26 |
| 3-class model | 17701.52 | 17225.32 |
| **4-class model** | **17748.15** | **17111.73** |
| 5-class model | 17880.73 | 17084.10 |
| 6-class model | 17979.14 | 17022.29 |

**Supplementary Table 4.** Observed/Expected ratio and Exclusivity ratio in the comorbidity and medication patterns

| Disease and Drug Groups | Observed/Expected Ratio | | | | Exclusivity | | | |
| --- | --- | --- | --- | --- | --- | --- | --- | --- |
|  | **Unspecific pattern** | **Diabetes and Liver pattern** | **Neurocognitive and Psychiatric pattern** | **Musculoskeletal, Immunological and Dermatological pattern** | **Unspecific pattern** | **Diabetes and Liver pattern** | **Neurocognitive and Psychiatric pattern** | **Musculoskeletal, Immunological and Dermatological pattern** |
| Antihypertensives | 1.03 | 1.06 | 0.91 | 0.97 | **40.3** | 15.7 | 12.8 | **31.2** |
| Lipid lowering agents | 0.91 | 1.34 | 0.83 | 1.02 | **35.7** | 19.9 | 11.6 | **32.8** |
| Cardiovascular/antiarrhythmics | 1.18 | 0.92 | 0.91 | 0.86 | **45.9** | 13.6 | 12.7 | **27.7** |
| Diuretics | 0.74 | 1.13 | 1.34 | 1.11 | **28.7** | 16.8 | 18.8 | **35.7** |
| Gastrointestinal therapeutics | 0.70 | 0.88 | 1.41 | 1.24 | **27.5** | 13.1 | 19.8 | **39.6** |
| Respiratory drugs | 0.12 | 0.82 | 1.39 | 1.98 | 4.9 | 12.2 | 19.5 | **63.4** |
| Anti-Parkinson drugs | 0.11 | 0.30 | **6.50** | 0.00 | 4.3 | 4.3 | **91.3** | 0.0 |
| Antiplatelets | 0.65 | 1.29 | 1.51 | 1.08 | **25.0** | 19.2 | 21.2 | **34.6** |
| Antidiabetics | 0.10 | **5.11** | 1.10 | 0.15 | 3.8 | **76.0** | 15.4 | 4.8 |
| Analgesics | 0.22 | 0.60 | 1.68 | 1.84 | 8.8 | 8.8 | 23.5 | **58.8** |
| Antigout agents | 1.21 | 1.59 | 0.69 | 0.61 | **47.2** | 23.6 | 9.7 | 19.4 |
| Endocrine drugs | 1.19 | 1.18 | 0.27 | 1.01 | **46.3** | 17.5 | 3.8 | **32.5** |
| Antiepileptics | 0.45 | 1.97 | **2.51** | 0.56 | 17.6 | **29.4** | **35.3** | 17.6 |
| Antipsychotics | 0.24 | 0.63 | **4.07** | 0.75 | 9.5 | 9.5 | **57.1** | 23.8 |
| Anti-inflammatory drugs | 0.08 | 0.40 | 0.00 | **2.84** | 3.0 | 6.1 | 0.0 | **90.9** |
| Antidepressants | 0.49 | 0.74 | **3.09** | 0.82 | 19.1 | 11.0 | **43.4** | **26.5** |
| Antiosteoporosis | 0.43 | 0.75 | 1.20 | 1.71 | 16.9 | 11.2 | 16.9 | **55.1** |
| Other drugs | 0.00 | 0.92 | **4.86** | 0.58 | 0.0 | 13.6 | **68.2** | 18.2 |
| Respiratory disease | 0.25 | 1.20 | 1.06 | 1.79 | 9.9 | 17.8 | 14.9 | **57.4** |
| Cardiovascular diseases | 0.93 | 1.06 | 1.00 | 1.06 | **36.2** | 15.8 | 14.1 | **33.9** |
| Endocrine & metabolic dis | 0.71 | 1.56 | 0.27 | 1.41 | **27.7** | 23.2 | 3.7 | **45.3** |
| Neurologic disease | 0.42 | 1.00 | **3.29** | 0.69 | 16.7 | 14.8 | **46.3** | 22.2 |
| Cognitive disease | 0.35 | 0.57 | **4.19** | 0.58 | 13.7 | 8.5 | **59.0** | 18.8 |
| Gastrointestinal disease | 0.47 | 0.92 | 0.82 | 1.76 | 18.2 | 13.6 | 11.6 | **56.6** |
| Hepatic disease | 0.17 | **2.14** | 0.32 | 1.77 | 6.8 | **31.8** | 4.5 | **56.8** |
| Genitourinary disease | 0.77 | 1.25 | 0.44 | 1.41 | **30.1** | 18.6 | 6.2 | **45.1** |
| Musculoskeletal disease | 0.37 | 0.67 | 0.78 | **2.01** | 14.5 | 9.9 | 11.0 | **64.5** |
| Diabetes | 0.18 | **4.91** | 1.10 | 0.15 | 7.0 | 72.9 | 15.5 | 4.7 |
| Haematologic disease | 0.19 | 1.50 | 1.18 | 1.68 | 7.4 | 22.2 | 16.7 | **53.7** |
| Immune disease | 0.29 | 0.66 | 0.00 | **2.45** | 11.5 | 9.8 | 0.0 | **78.7** |
| Dermatological disease | 0.22 | 0.71 | 0.75 | **2.20** | 8.5 | 10.6 | 10.6 | **70.2** |
| Cancer | 0.73 | 0.86 | 0.92 | 1.42 | **28.7** | 12.9 | 12.9 | **45.5** |
| Chronic kidney failure | 0.81 | 1.56 | 1.30 | 0.83 | **31.7** | 23.2 | 18.3 | **26.8** |
| Other chronic diseases | 0.23 | 1.36 | 0.64 | 1.93 | 9.0 | 20.2 | 9.0 | **61.8** |
| Other neurologic disease | 0.98 | **2.99** | 0.00 | 0.53 | **38.9** | **44.4** | 0.0 | 16.7 |

Bold values indicate O/E ≥ 2 and exclusivity ≥ 25%. The drug and disease groups were assigned to latent classes when both thresholds obtained.

**Supplementary Table 5.** Adjusted hazard ratios of subgroup analysis stratified by sex, age, mobility status, and presence of oncologic diseases and multiplicative interaction

|  | Stratified by Sex | | | | | | | | | | | Interaction  p value |
| --- | --- | --- | --- | --- | --- | --- | --- | --- | --- | --- | --- | --- |
| Outcomes | **Men** | **HR** | **95% CI** | | | **p value** | **Women** | **HR** | **95% CI** | | **p value** |  |
| Composite Outcome | UN | ref | ref | ref | ref | | UN | ref | ref | ref | ref | ref |
|  | DL | 1.09 | 0.48 | 2.47 | 0.828 | | DL | 2.29 | 1.06 | 4.95 | **0.036** | 0.195 |
|  | NCP | 1.33 | 0.66 | 2.71 | 0.423 | | NCP | 2.38 | 1.15 | 4.90 | **0.019** | 0.262 |
|  | MID | 1.15 | 0.62 | 2.13 | 0.658 | | MID | 1.54 | 0.79 | 2.99 | 0.207 | 0.530 |
| Thromboembolic outcome | UN | ref | ref | ref | ref | | UN | ref | ref | ref | ref | ref |
|  | DL | 1.14 | 0.22 | 5.91 | 0.873 | | DL | 1.74 | 0.42 | 7.20 | 0.443 | 0.698 |
|  | NCP | 1.38 | 0.27 | 6.93 | 0.696 | | NCP | 5.14 | 1.68 | 15.66 | **0.004** | 0.188 |
|  | MID | 1.48 | 0.44 | 4.92 | 0.525 | | MID | 2.04 | 0.71 | 5.87 | 0.184 | 0.690 |
| Bleeding outcome | UN | ref | ref | ref | ref | | UN | ref | ref | ref | ref | ref |
|  | DL | 2.09 | 0.68 | 6.38 | 0.196 | | DL | 1.97 | 0.43 | 8.98 | 0.383 | 0.951 |
|  | NCP | 2.19 | 0.69 | 6.96 | 0.184 | | NCP | 3.23 | 0.79 | 13.14 | 0.102 | 0.675 |
|  | MID | 2.43 | 0.96 | 6.12 | 0.060 | | MID | 1.89 | 0.55 | 6.55 | 0.315 | 0.752 |
| Fall outcome | UN | ref | ref | ref | ref | | UN | ref | ref | ref | ref | ref |
|  | DL | 1.26 | 0.65 | 2.42 | 0.498 | | DL | 0.87 | 0.45 | 1.69 | 0.681 | 0.439 |
|  | NCP | 1.01 | 0.52 | 1.97 | 0.978 | | NCP | 0.83 | 0.44 | 1.58 | 0.577 | 0.684 |
|  | MID | 0.92 | 0.53 | 1.59 | 0.756 | | MID | 1.29 | 0.82 | 2.03 | 0.266 | 0.343 |
|  | **Stratified by Mobility** | | | | | | | | | | | **Interaction  p value** |
| Outcomes | **Dependent patients** | **HR** | **95% CI** | | | **p value** | **Independent patients** | **HR** | **95% CI** | | **p value** |  |
| Composite Outcome | UN | ref | ref | ref | ref | | UN | ref | ref | ref | ref | ref |
|  | DL | 2.22 | 0.91 | 5.44 | 0.081 | | DL | 1.28 | 0.61 | 2.68 | 0.520 | 0.350 |
|  | NCP | 2.59 | 1.27 | 5.30 | **0.009** | | NCP | 0.94 | 0.36 | 2.47 | 0.895 | **0.100** |
|  | MID | 1.95 | 0.89 | 4.27 | 0.097 | | MID | 1.12 | 0.64 | 1.97 | 0.687 | 0.265 |
| Thromboembolic outcome | UN | ref | ref | ref | ref | | UN | ref | ref | ref | ref | ref |
|  | DL | 0.88 | 0.08 | 9.95 | 0.915 | | DL | 1.66 | 0.50 | 5.50 | 0.408 | 0.643 |
|  | NCP | 5.19 | 1.08 | 24.90 | **0.039** | | NCP | 1.56 | 0.34 | 7.27 | 0.568 | 0.285 |
|  | MID | 4.43 | 0.91 | 21.65 | 0.066 | | MID | 1.11 | 0.41 | 2.95 | 0.842 | 0.144 |
| Bleeding outcome | UN | ref | ref | ref | ref | | UN | ref | ref | ref | ref | ref |
|  | DL | 6.29 | 0.65 | 60.96 | 0.113 | | DL | 1.81 | 0.62 | 5.26 | 0.276 | 0.331 |
|  | NCP | 10.99 | 1.34 | 90.19 | **0.026** | | NCP | 1.13 | 0.24 | 5.30 | 0.874 | **0.088** |
|  | MID | 5.41 | 0.60 | 48.89 | 0.133 | | MID | 2.12 | 0.93 | 4.84 | 0.075 | 0.434 |
| Fall outcome | UN | ref | ref | ref | ref | | UN | ref | ref | ref | ref | ref |
|  | DL | 1.13 | 0.54 | 2.39 | 0.742 | | DL | 1.14 | 0.61 | 2.10 | 0.682 | 0.995 |
|  | NCP | 0.64 | 0.31 | 1.29 | 0.213 | | NCP | 1.97 | 1.02 | 3.79 | **0.044** | **0.023** |
|  | MID | 1.48 | 0.81 | 2.72 | 0.203 | | MID | 1.17 | 0.75 | 1.81 | 0.489 | 0.529 |
|  | **Stratified by Age (cut off 80 years)** | | | | | | | | | | | **Interaction  p value** |
| Outcomes | **Age ≥80 years** | **HR** | **95% CI** | | | **p value** | **Age <80 years** | **HR** | **95% CI** | | **p value** |  |
| Composite Outcome | UN | ref | ref | ref | ref | | UN | ref | ref | ref | ref | ref |
|  | DL | 1.40 | 0.69 | 2.85 | 0.351 | | DL | 1.84 | 0.74 | 4.57 | 0.186 | 0.639 |
|  | NCP | 1.78 | 1.03 | 3.07 | **0.040** | | NCP | 1.06 | 0.24 | 4.79 | 0.936 | 0.529 |
|  | MID | 1.12 | 0.64 | 1.95 | 0.701 | | MID | 1.72 | 0.79 | 3.72 | 0.171 | 0.375 |
| Thromboembolic outcome | UN | ref | ref | ref | ref | | UN | ref | ref | ref | ref | ref |
|  | DL | 0.46 | 0.06 | 3.85 | 0.477 | | DL | 2.43 | 0.63 | 9.34 | 0.197 | 0.191 |
|  | NCP | 3.19 | 1.20 | 8.51 | **0.020** | | NCP | na | na | na | na | 1.000 |
|  | MID | 1.54 | 0.55 | 4.36 | 0.412 | | MID | 1.92 | 0.58 | 6.33 | 0.286 | 0.787 |
| Bleeding outcome | UN | ref | ref | ref | ref | | UN | ref | ref | ref | ref | ref |
|  | DL | 1.73 | 0.56 | 5.34 | 0.339 | | DL | 4.50 | 0.81 | 24.93 | 0.085 | 0.358 |
|  | NCP | 1.46 | 0.54 | 3.98 | 0.455 | | NCP | 6.47 | 0.90 | 46.45 | 0.063 | 0.187 |
|  | MID | 1.49 | 0.62 | 3.59 | 0.374 | | MID | 5.42 | 1.15 | 25.64 | 0.033* | 0.156 |
| Fall outcome | UN | ref | ref | ref | ref | | UN | ref | ref | ref | ref | ref |
|  | DL | 1.44 | 0.80 | 2.62 | 0.226 | | DL | 0.77 | 0.36 | 1.65 | 0.499 | 0.197 |
|  | NCP | 1.17 | 0.69 | 1.97 | 0.567 | | NCP | 0.49 | 0.12 | 2.09 | 0.338 | 0.273 |
|  | MID | 1.54 | 0.98 | 2.40 | 0.059 | | MID | 0.88 | 0.49 | 1.59 | 0.682 | 0.138 |
|  | **Stratified by cancer status** | | | | | | | | | | | **Interaction  p value** |
| Outcomes | **Cancer** | **HR** | **95% CI** | | | **p value** | **No cancer** | **HR** | **95% CI** | | **p value** |  |
| Composite Outcome | UN | ref | ref | ref | ref | | UN | ref | ref | ref | ref | ref |
|  | DL | 0.99 | 0.11 | 8.71 | 0.990 | | DL | 1.63 | 0.92 | 2.90 | 0.097 | 0.661 |
|  | NCP | 2.96 | 0.78 | 11.26 | 0.111 | | NCP | 1.70 | 0.99 | 2.93 | 0.056 | 0.451 |
|  | MID | 1.46 | 0.48 | 4.39 | 0.504 | | MID | 1.29 | 0.78 | 2.13 | 0.326 | 0.843 |
| Thromboembolic outcome | UN | ref | ref | ref | ref | | UN | ref | ref | ref | ref | ref |
|  | DL | 3.15 | 0.19 | 51.84 | 0.421 | | DL | 1.11 | 0.34 | 3.61 | 0.861 | 0.498 |
|  | NCP | 4.09 | 0.25 | 67.97 | 0.326 | | NCP | **2.93** | **1.18** | **7.25** | **0.020** | 0.823 |
|  | MID | 3.81 | 0.44 | 33.03 | 0.225 | | MID | 1.37 | 0.56 | 3.36 | 0.493 | 0.389 |
| Bleeding outcome | UN | ref | ref | ref | ref | | UN | ref | ref | ref | ref | ref |
|  | DL | 0.56 | 0.06 | 5.16 | 0.610 | | DL | **2.95** | **1.05** | **8.26** | **0.039** | 0.181 |
|  | NCP | na | na | na | na | | NCP | **4.14** | **1.52** | **11.26** | **0.005** | na |
|  | MID | 0.38 | 0.08 | 1.74 | 0.213 | | MID | **3.48** | **1.43** | **8.46** | **0.006** | **0.014** |
| Fall outcome | UN | ref | ref | ref | ref | | UN | ref | ref | ref | ref | ref |
|  | DL | 1.51 | 0.30 | 7.65 | 0.620 | | DL | 1.06 | 0.65 | 1.73 | 0.814 | 0.682 |
|  | NCP | 0.99 | 0.20 | 4.81 | 0.988 | | NCP | 0.95 | 0.58 | 1.57 | 0.851 | 0.967 |
|  | MID | 1.87 | 0.73 | 4.75 | 0.191 | | MID | 1.19 | 0.80 | 1.76 | 0.386 | 0.384 |

Bold values depict statistically significant results at p < 0.05 level. Interaction p value of ≤ 0.10 suggests a potential significancy or association that may require further investigation. CI: confidence Interval. Dependent category included bedridden, walker, and wheelchair users due to the insufficient number of patients for the computation of these levels separately. Na: not applicable due to sample size. UN: Unspecific pattern, DL: Diabetes and liver pattern, NCP: Neurocognitive and psychiatric pattern, MID: Musculoskeletal, immunologic and dermatologic pattern. The number of patients with cancer in each pattern is: UN 29, DL 13, NCP 13, and MID 46.

**Supplementary Table 6.** Adjusted hazard ratios for adverse clinical outcomes stratified by cancer status of AF patients

|  | Stratified by cancer status | | | | | | | | | | |
| --- | --- | --- | --- | --- | --- | --- | --- | --- | --- | --- | --- |
| Outcomes | **Cancer** | **HR** | **95% CI** | | | **p value** | **No cancer** | **HR** | **95% CI** | | **p value** |
| Composite Outcome | UN | ref | ref | ref | ref | | UN | ref | ref | ref | ref |
|  | DL | 1.01 | 0.09 | 10.69 | 0.996 | | DL | 1.64 | 0.92 | 2.92 | 0.095 |
|  | NCP | 2.94 | 0.74 | 11.69 | 0.126 | | NCP | 1.71 | 0.99 | 2.95 | 0.055 |
|  | MID | 1.91 | 0.61 | 5.96 | 0.264 | | MID | 1.30 | 0.78 | 2.15 | 0.309 |
| Thromboembolic outcome | UN | ref | ref | ref | ref | | UN | ref | ref | ref | ref |
|  | DL | 0.41 | 0.03 | 5.26 | 0.490 | | DL | 2.61 | 0.92 | 7.41 | 0.072 |
|  | NCP | na | na | na | na | | NCP | **3.50** | **1.27** | **9.62** | **0.015** |
|  | MID | 0.20 | 0.03 | 1.31 | 0.090 | | MID | **3.59** | **1.47** | **8.74** | **0.005** |
| Bleeding outcome | UN | ref | ref | ref | ref | | UN | ref | ref | ref | ref |
|  | DL | 0.64 | 0.05 | 7.51 | 0.722 | | DL | **3.05** | **1.09** | **8.55** | **0.033** |
|  | NCP | na | na | na | na | | NCP | **4.21** | **1.54** | **11.47** | **0.005** |
|  | MID | 0.57 | 0.12 | 2.85 | 0.496 | | MID | **3.62** | **1.48** | **8.84** | **0.005** |
| Fall outcome | UN | ref | ref | ref | ref | | UN | ref | ref | ref | ref |
|  | DL | 3.15 | 0.49 | 20.08 | 0.226 | | DL | 1.03 | 0.63 | 1.69 | 0.910 |
|  | NCP | 1.49 | 0.28 | 7.88 | 0.641 | | NCP | 0.97 | 0.59 | 1.60 | 0.910 |
|  | MID | 1.90 | 0.70 | 5.16 | 0.207 | | MID | 1.23 | 0.83 | 1.82 | 0.305 |

Bold values depict statistically significant results at p < 0.05 level. CI: confidence Interval. Na: not applicable due to small sample size. UN: Unspecific pattern, DL: Diabetes and liver pattern, NCP: Neurocognitive and psychiatric pattern, MID: Musculoskeletal, immunological and dermatological pattern.
